# TNF-α − 308 G/A and IFN-γ + 874 A/T gene polymorphisms in Saudi patients with cutaneous leishmaniasis

**DOI:** 10.1186/s12881-020-01043-9

**Published:** 2020-05-13

**Authors:** Ahmed A. Ahmed, Zafar Rasheed, Tarek Salem, Mohammed S. Al-Dhubaibi, Ahmad A. Al Robaee, Abdullateef A. Alzolibani

**Affiliations:** 1 Research Center, College of Medicine, Qassim University, Buraidah, Saudi Arabia; 2 Department of Medical Biochemistry, College of Medicine, Qassim University, P.O. Box 6655, Buraidah, KSA 51452 Saudi Arabia; 3 Department of Dermatology, College of Medicine, Qassim University, Buraidah, Saudi Arabia

**Keywords:** Cutaneous leishmaniasis, Gene polymorphism, TNF-α, IFN-γ, *L. major*, *L. tropica*

## Abstract

**Background:**

Cutaneous leishmaniasis (CL) is well linked with immunogenetic factors. This study was undertaken to test the association of TNF-α − 308 and IFN-γ + 874 gene polymorphisms with the susceptibility of *Leishmania (L) species* among CL patients in central region of Saudi Arabia.

**Methods:**

This is a case-control study involved 169 Saudi subjects with different *L. species* and 199 healthy controls from central region of Saudi Arabia. All subjects were characterized by TNF-α − 308 G/A and IFN-γ + 874 A/T gene polymorphisms using PCR.

**Results:**

Evaluation of genotyping and allelic frequency of TNF-α − 308 G/A in different *L. species* showed no significant association compared to controls (*p* > 0.05). Except, in cases of *L. tropica* that showed significantly higher TNF-α − 308 A versus G allele frequency (*p* = 0.0004). Evaluation of genotyping of IFN-γ + 874 (TT versus AA+AT recessive) and allelic frequency of IFN-γ + 874 (T versus A) showed significant higher in *L. major* and also in total CL cases as compared to healthy controls (*p* < 0.05). Furthermore, a strong association was observed between the susceptibility of *L. major*, *L. tropica or* total CL cases with synergistically combined high TNF-α 308/INF-γ 874 alleles.

**Conclusions:**

This is the first report that shows the gene polymorphisms of TNF-α − 308 G/A and IFN-γ + 874 A/T in Saudi patients with different *L. species* infections*.* Data showed that the TNF-α-308 G/A gene polymorphism is not associated with the susceptibility of CL in Saudi subjects. The only correlation was found in between A versus G allelic frequency in *L. tropica.* Importantly, IFN-γ + 874 A/T polymorphism was found to be associated with the susceptibility of *L. major* and also with total CL subjects. Moreover, data from synergistically combined high TNF-α 308/INF-γ 874 alleles strongly suggest their potential role in the susceptibility of leishmania infection.

## Background

*Leishmania* is a one of the most common forms of trypanosomiasis protozoan, which is endemic throughout the tropical and subtropical regions of the globe [1]. Cutaneous leishmaniasis (CL) is the most frequent *leishmania* infection, known to induce dermal lesions that most likely produce permanent life-long scars. Now it is well known that CL infection is primarily caused by the transmitting the parasites from the female phlebotomine sandflies bite [1]. Etiology of CL infection dependents on the number of factors such as the characteristics of the parasite, type of sand-fly species, the ecological regions, current/former exposure of infection, and also on the human behavior [2, 3]. It is now well documented that the *leishmania* has more than 22 different species but their prevalence varies from region to region [3]. These *L. species* have a wide geographic distribution from South/North America to all over Middle East with a significant increase of number of patients in last few years in the countries such as Bolivia, Brazil, Peru, Colombia, Afghanistan, Iran, Syria, Algeria and also in Saudi Arabia [2–5]. Furthermore, the distribution of *L. species* or CL also reported to have an association with the number of environmental factors including temperature and humidity. Moreover, the urbanization and migration have also been documented to have an association with the global diffusion of *leishmania* infection [1, 4]. In Saudi Arabia, despite of the several important efforts taken by the government, but CL remains to be a major heath issue of the country. Several studies have shown that the *L. major* and *L. tropica* are the principal reservoirs of this infection, as these species are distributed in almost all region of the country [5]. Reports have also shown that the phlebotomine sandflies are commonly found in the desert regions around the farms, which assumed to be one of the responsible factors for the exposure of this parasitic infection among Saudi population [5].

It is now well established that the proinflammatory cytokines play several crucial roles in onset of CL, which varies from the resistance or the host immune response to infection, especially tumor necrosis factor alpha (TNF-α) and interferon-gamma (IFN-γ) [6]. The role of TNF-α and IFN-γ was well reported in *L. major* as both of these cytokines are produced by T helper 1 (Th1) cells, which are synergistically activates macrophages [7]. Furthermore, an association of TNF-αor IFN-γ with the increase of tissue damage or ulceration was also reported in CL patients [8]. Moreover, the role of Th1 response against *L. major* infection was also demonstrated in animal model of *L. major* [9, 10]. Out of all studied Th1 cytokines, TNF-α and IFN-γ were found to be critical for the initiation of preventive immunity against all studied *L. species,* particularly *L. major* [10–13]. TNF-α-308G/A polymorphism was well studied in several autoimmune/inflammatory diseases, the A allele of TNFα (308G/A) was reported to have higher transcriptional activity by 6–7 folds as compared with the common G allele [14, 15]. Therefore, the A allele frequency has been implicated in the pathogenesis of several infectious as well as autoimmune disorders [14, 15]. On the other hand, IFN-γ-874 A/T polymorphism was also reported to have a genetic link with several autoimmune/inflammatory disorders through + 874 T allele carriers [16, 17]. However, it is still unclear whether TNF-α − 308 G/A or IFN-γ + 874 A/T polymorphisms play a role in the *leishmania* infection.

In view of these, we hypothesized that TNF-α-308 G/A and IFN-γ + 874 A/T gene polymorphisms may be associated with *L. species* infection. To test this hypothesis, DNA samples were isolated from CL patients and the association between TNF-α-308 G/A or IFN-γ + 874 A/T gene polymorphisms with the *L. species* susceptibility was studied.

## Methods

### Human subjects

The study was carried out in accordance with the Code of Ethics of the World Medical Association (Declaration of Helsinki as revised in Tokyo 2004) for humans and the study protocol was approved by National Plan for Science, Technology and Innovation of Saudi Arabia (NSTIP/KACST # 11-MED1068–09). With the Institutional Review Board (IRB) approval, the participants were recruited from the clinical units and the Qassim hospitals, KSA. All patients were recruited after thorough examination based on the patients clinical appearance, microscopic and specific PCR-based observations as described previously [18, 19]. Briefly, patients with compatible lesions (range 1–14) on limb, facial or skull were included. Patients were also screened by the parasitological examination of *L. species* infection by culture, histological and DNA based PCR examination. Exclusion criteria of the patient’s selection were: (i) CL patients co-infected with other pathogen/infection; (ii) patients having history of other parasitic/viral infection; (iii) less than 18 years old; (iv) current pregnancy or lactation. Samples from normal healthy controls were also collected from the same Qassim hospitals, all selected control humans showed negative tests for all infections including CL. After thorough clinical and molecular examination, 169 adult with CL (91 males; 78 females; age 34.1 ± 3.5 years) and 199 healthy subjects (106 males and 93 females, 32.8 ± 7.1 years) were included in this study (Supplementary file). The complete demographic and clinical findings of studied cutaneous leishmaniasis patients are summarized in Table 1.

**Table 1:** Demographic details of studied cutaneous leishmaniasis subjects

Parameters	Results
Total number of studied *leishmania* subjects	169
Patients age (mean ± SD., years)	34.1 ± 3.5
Gender	91 males; 78 females
Disease duration (mean ± SD., months)	55.91 ± 34.7
Sites of lesions	Limb, facial or skill
No. of lesions	1–14
*Leishmania major*	102
*Leishmania tropica*	59
*Leishmania infantum/donovani complex*	08
Total number of healthy human controls	199
Age of controls (mean ± SD., years)	36.8 ± 7.2
Gender of controls	106 males; 93 females

### DNA extraction and amplification

Genomic DNA was extracted from the lymphocytes of peripheral blood from the patients and controls by the MagNA Pure LC instrument (Roche Diagnostics GmbH, Roche Molecular Biochemical, Mannheim, Germany) as described previously [20]. The purity and quantity of DNA was measured the by absorbance ration 260/280 as described previously [21]. The genetic variants of TNF-α 308 G/A (rs1800629), and IFN-γ 874 T/A (rs2430561) polymorphisms were amplified by the PCR based Amplification Refractory Mutation System (ARMS-PCR) using the primers sequence summarized in Table 2. The ARMS-PCR for the TNF-α − 308 G/A gene polymorphism was performed two times for all subjects including patients and healthy human controls. One time the ARMS-PCR was performed by using common forward primer (5`-TCTCGGTTTCTTCTCCATCG-3`) and reverse primer represented for G genotype (5`-ATAGGTTTTGAGGGGCATGG-3) and the second PCR was performed by the same common primer with the reverse primer that represented A genotype (5`-ATAGGTTTTGAGGGGCATGA-3`). Detection at the correct size band with reverse primer G that means the homozygote genotype GG, whereas detection of the same size band when repeated with reverse primer A that means the homozygote genotype AA. If detected the correct band from the first run with G and also with the second run with A for the same patients, that means the genotype represents heterozygote GA. Similarly, the IFN-γ + 874 A/T gene polymorphism was validated by calculation homozygote TT or GG alleles and heterozygote TA allele. The PCR amplification was carried out using Primus HT Dual Thermal Cycler PCR (MWG AG Biotech, USA) in a 25 μl total volume containing about 100 ng of genomic DNA template, 1 μM of each primer, 1 × Go Taq® green master mix (Promega). The initial denaturation in PCR was carried out at 95 °C for 2 min followed by 30 cycles of 95 °C for 15 s, 65 °C for 50 s, and 72 °C for 60s. After PCR running, all amplified PCR products were electrophoresed on 2% agarose gel and PCR products were visualized under UV light using ethidium bromide stain.

**Table 2:** Primer sequences used for TNF-α A/G and INF-γ T/A genotypes and allelic polymorphisms in skin biopsies of leishmaniasis patients

**SNo.**	**TNF-α-308 (rs1800629)**	
1	Forward Primer (Common)	5`-TCTCGGTTTCTTCTCCATCG-3`
2	Reverse Primer 1 (G allele)	5`-ATAGGTTTTGAGGGGCATGG-3
3	Reverse Primer 2 (A allele)	5`-ATAGGTTTTGAGGGGCATGA-3`
**SNo.**	**INF-γ-874 (rs2430561)**	
1	Forward Primer (Common)	5`-TCAACAAAGCTGATACTCCA-3`
2	Reverse Primer 1 (T allele)	5`-TTCTTACAACACAAAATCAAATCT-3
3	Reverse Primer 2 (A allele)	5`-TTCTTACAACACAAAATCAAATCA-3

### Statistical analysis

Data were analyzed using the statistical software program SPSS version 17. The frequencies of studied genotypic and allelic polymorphisms among cases were compared to those of controls using Fisher’s exact test and odds ratio (OR) with the 95% confidence interval (95% CI). A *p* level of < 0.05 was considered significant.

## Results

**Fig. 1:**
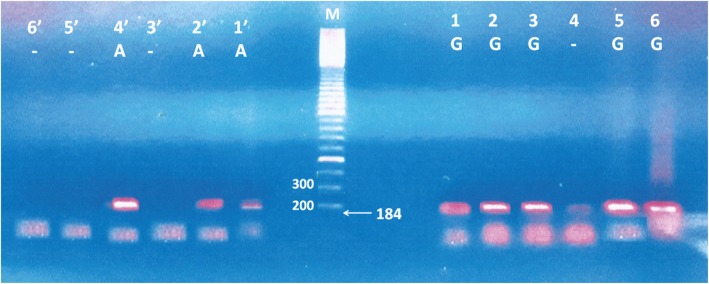
TNF-α − 308 G/A polymorphism in CL patients. Lane M indicates for DNA ladder 100 bp, lanes 1 and 1’represent GA genotype for sample 1, lanes 2 and 2` represent GA for sample 2, lanes 3 and 3` represent GG genotype for sample 3, lanes 4 and 4` represent AA genotype for sample 4, lanes 5 and 5` represent GG genotype for sample 5 and lanes 6 and 6` represent GG genotype for sample 6. All TNF-α − 308 G/A genotype polymorphism detected at the same size (184 bp) by the amplification refractory mutation system

The amplified PCR product for TNF-α-308 was detected at 184 base pair as shown in Fig. 1, based on these results, different species of CL, the genotypes and the alleles of the host genes polymorphism were determined and evaluated in comparison with their respective healthy controls (supplementary file). Our data showed that the host TNF-α (GA + AA genotypes) versus those with GG genotype (dominant model) revealed no significant association with *L. major, L. tropica* and total number of CL cases as compared with their respective controls [*p* = 0.3, OR (95%CI) 1.31 (0.8 to 2.2), *p* = 0.4, OR (95%CI) 1.3 (0.7 to 2.3), and *p* = 0.2, OR (95%CI) 1.3 (0.9 to 2.0), respectively] (Table 3). TNF-α AA versus GG + GA genotypes (recessive model) revealed also no significant association with *L. major, L. tropica* and total number of CL cases as compared with their respective controls [*p* = 0.6, OR (95%CI) 1.5 (0.3 to 6.7), *p* = 0.5 OR (95%CI) 0.4 (0.02 to 0.7) and *p* = 0.2 OR (95%CI) 1.3 (0.9 to 2.0) respectively] (Table 3). On the other hand allelic frequency of A versus G was revealed no significant difference between *L. major* and control [*p* = 0.3, OR (95%CI) 1.3 (0.8 to 1.9)] (Table 3). In contrast to *L. tropica* showed significant association with A versus G allele frequency [*p* = 0.0004, OR (95%CI) 2.2 (1.4 to 3.4)]. Whereas, the total number of different species also showed no significant association [*p* = 0.3, OR (95%CI) 1.2 (0.8 to 1.7)]. The complete details of TNF-α 308 G/A polymorphism in different *L. species* and healthy human controls are summarized in Table 3.

**Table 3:** Genotype frequencies of TNF-α-308 G/A in different species of leishmaniasis and healthy human controls

**TNF-α 308 G/A**	***L. major*** **102 (%)**	**Healthy human controls** **199 (%)**
Genotypes		
GG	60 (58.8)	130 (65.3)
GA	39 (38.2)	65 (32.7)
AA	3 (3.0)	4 (2.0)
Alleles		
G	159 (78)	325 73 (81.7)
A	45 (22)	73 (18.3)
Statistics		
AA+GA vs. GG (dominant)	*P* = 0.3	OR (95%CI) 1.31 [0.8 to 2.2]
AA vs. GG + GA (recessive)	*P* = 0.6	OR (95%CI) 1.5 [0.3 to 6.7]
A allele vs. G allele	*P* = 0.3	OR (95%CI) 1.3 [0.8 to 1.9]
**TNF-α 308 G/A**	***L. tropica*** **59 (%)**	**Healthy human controls** **199 (%)**
Genotypes		
GG	35 (59.3)	130 (65.3)
GA	24 (40.7)	65 (32.7)
AA	0 (0)	4 (2)
Alleles		
G	91 (79.6)	325 (81.7)
A	27 (20.4)	73 (18.3)
Statistics		
AA+GA vs. GG (dominant)	*P* = 0.4	OR (95%CI) 1.3 [0.7 to 2.3]
AA vs. GG + GA (recessive)	*P* = 0.5	OR (95%CI) 0.4 [0.02 to 7.1]
A allele vs. G allele	P** =0.0004	OR (95%CI) 2.2 [1.4 to 3.4]
**TNF-α 308 G/A**	***L. infantum/donovani complex*** **8 (%)**	**Healthy human controls** **199 (%)**
Genotypes		
GG	5 (62.5)	130 (65.3)
GA	3 (37.5)	65 (32.7)
AA	0 (0)	4 (2)
Alleles		
G	13 (77.4)	325 (81.7)
A	3 (22.6)	73 (18.3)
Statistics		
ND	ND	ND
**TNF-α 308 G/A**	**Total*leishmania*cases** **169 (%)**	**Healthy human controls** **199 (%)**
Genotypes		
GG	100 (59.2)	130 (65.3)
GA	66 (39.1)	65 (32.7)
AA	3 (1.7)	4 (2)
Alleles		
G	266 (78.7)	325 (81.7)
A	72 (21.3)	73 (18.3)
Statistics		
AA+GA vs. GG (dominant)	*P* = 0.2	OR (95%CI) 1.3 [0.9 to 2.0]
AA vs. GG + GA (recessive)	*p* = 0.9	OR (95%CI) 0.9 [0.2 to 4.0]
A alleles vs. G allele	*p* = 0.3	OR (95%CI) 1.2 [0.8 to 1.7]

P*significant

P** highly significant

**Fig. 2:**
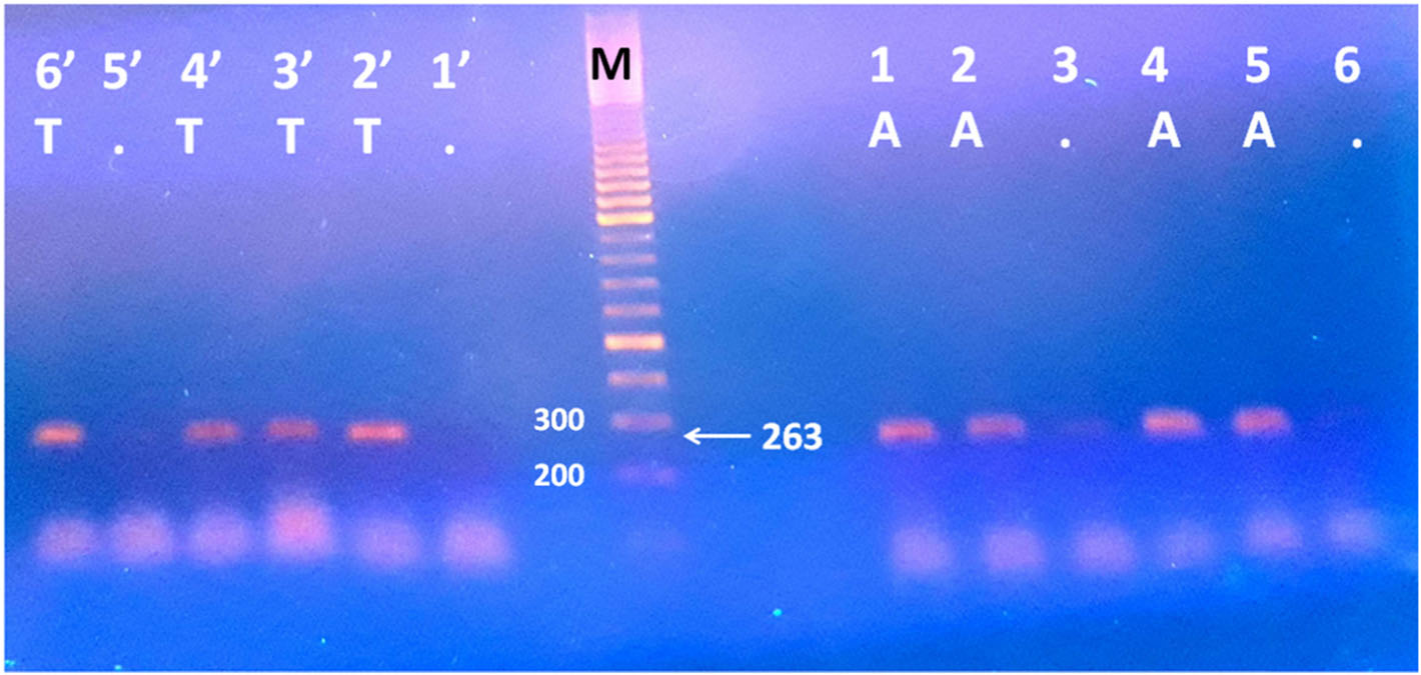
IFN-γ + 874 A/T polymorphism in CL patients. Lane M indicate DNA ladder 100 bp, lanes 1 and 1’represent AA genotype for sample 1, lanes 2 and 2` represent AT genotype for sample 2, lanes 3 and 3` represent TT genotype for sample 3, lanes 4 and 4` represent AT genotype for sample 4, lanes 5 and 5` represent AA genotype for sample 5 and lanes 6 and 6` represent TT genotype for sample 6. All IFN-γ +874 A/T genotypes detected at the same size (263 bp) by the amplification refractory mutation system

The amplified PCR product for IFN-γ + 874 were detected at 263 base pair as shown in Fig. 2, based on these results, different species of CL, the genotypes and the alleles of the host genes polymorphism were determined and evaluated in comparison with their respective healthy controls. Results show in Table 4, pointed out that the host INF-γ + 874 (TT + AT genotypes) versus those with the AA genotype (dominant model) revealed no significant association with *L. major, L. tropica* and total number CL cases in comparison with their respective controls [*p* = 0.2, OR (95% CI) 1.4 (0.8 to 2.4), *p* = 0.3, OR (95% CI) 1.4 (0.8 to 2.6), and *p* = 0.06 OR (95% CI) 1.5 (1.0 to 2.3), respectively]. On the contrary, host INF-γ + 874 (TT versus AA+AT genotypes) (recessive model) revealed high significant association with *L. major* and the total number CL cases as compared with controls [*p* = 0.02, OR (95%CI) 2.7 (1.2 to 5.9), and *p* = 0.01 OR (95% CI) 2.5 (1.2 to 5.3), respectively. Whereas with *L. tropica* no significant association was found [*p* = 0.06 OR (95%CI) 2.4 (0.9 to 6.2)]. In the other hand allelic frequency of T versus A revealed a significant association with *L. major* and also with total number of CL patients as compared with their respective controls [*p* = 0.02, OR (95%CI) 1.5 (1.07 to 2.13 and *p* = 0.02, OR (95%CI) 1.4 (1.07 to 2.0), respectively]. In contrast to these, *L. tropica* demonstrated no significant association with T versus A allele frequency [*p* = 0.1, OR (95% CI) 1.4 (0.9 to 2.1)]. Patients with *L. infantum/donavani* complex showed no statistical significant association with the INF-γ + 874 A/T polymorphism as the number of tested samples for this *L. species* was relatively low number (8 cases only). The complete details of INF-γ + 874 A/T polymorphism for all studied subjects are summarized in Table 4.

**Table 4:** Genotype frequencies of INF-γ + 874 A/T in different species of leishmaniasis and healthy human controls

**INF-γ 874 A/T**	***L. major*** **102 (%)**	**Healthy human controls** **199 (%)**
Genotypes		
AA	28 (27.5)	76 (38.2)
AT	59 (57.8)	111 (55.8)
TT	15 (14.7)	12 (6.0)
Alleles		
A	115 (56.4)	263 (66.1)
T	89 (43.6)	135 (33.9)
Statistics		
TT + AT vs. AA (dominant)	*P* = 0.2	OR (95%CI)1.4 [0.8 to 2.4]
TT vs. AA+AT (recessive)	P* = 0.02	OR (95%CI) 2.7 [1.2 to 5.9]
T allele vs. A allele	p* = 0.02	OR (95%CI)1.5 [1.07 to2.13]
**INF-γ 874 A/T**	***L. tropica*** **59 (%)**	**Healthy human controls** **199 (%)**
Genotypes		
AA	18 (30.5)	76 (38.2)
AT	33 (56)	111 (55.8)
TT	8 (13.5)	12 (6.0)
Alleles		
A	69 (58.5)	263 (66.1)
T	49 (41.5)	135 (33.9)
Statistics		
TT + AT vs. AA (dominant)	*P* = 0.3	OR (95%CI)1.4 [0.8 to 2.6]
TT vs. AA+AT (recessive)	*p* = 0.06	OR (95%CI) 2.4 [0.9 to 6.2]
T allele vs. A allele	*p* = 0.1	OR (95%CI)1.4 [0.9 to 2.1]
**INF-γ 874 A/T**	***L. infantum/ donovani complex*** **8 (%)**	**Healthy human controls** **199 (%)**
Genotypes		
AA	3 (37.5)	76 (38.2)
AT	4 (50)	111 (55.8)
TT	1 (12.5)	12 (6.0)
Alleles		
A	10 (62.5)	263 (66.1)
T	6 (37.5)	135 (33.9)
Statistics		
NA	NA	NA
**INF-γ 874 A/T**	**Total*leishmania*** **169(%)**	**Healthy human controls** **199 (%)**
Genotypes		
AA	49 (29)	76 (38.2)
AT	96 (56.8)	111 (55.8)
TT	24 (14.2)	12 (6.0)
Alleles		
A	194 (57.4)	263 (66.1)
T	144 (42.6)	135 (33.9)
Statistics		
TT + AT vs. AA (dominant)	*P* = 0.06	OR (95%CI) 1.5 [1.0 to 2.3]
TT vs. AA+AT (recessive)	p* = 0.01	OR (95%CI) 2.5 [1.2 to 5.3]
T allele vs. A allele	p* = 0.02	OR (95%CI) 1.4 [1.07 to2.0]

P* significant

P** highly significant

To further re-evaluate the potential role of genotype frequencies of TNF-α 308 G/A and INF-γ + 874 A/T in the susceptibility of CL infection among the studied Saudi patients, the data were analyzed by the synergistically combined genotype frequencies of TNF-α 308 G/A and INF-γ + 874 A/T in all CL patients and normal human controls. Analysis from the synergistically combined TNF-α 308 G/A and INF-γ + 874 A/T genotype showed a stronger association of the high TNF-α/INF-γ genotype with the susceptibility of *L. major* [*p* = 0.005 OR (95% CI) 2.2(1.2 to 3.8)], *L. tropica* [*p* = 0.003, OR (95% CI) 2.7(1.4 to 5.1)] and also with total studied CL patients [(*p* = 0.001, OR (95% CI) 2.2(1.2 to 3.7)] respectively. The complete details of the synergistically combined genotype frequencies of TNF-α-308 G/A and INF-γ 874 A/T in all studied *L. species* and human controls are summarized in Table 5. These results further support a potential role of TNF-α-308 G/A and INF-γ 874 A/T in the pathogenesis of CL infection among the studied Saudi population.

**Table 5:** Synergistically combined genotype frequencies of TNF-α-308 G/A and INF-γ 874 A/T in different species of leishmaniasis and healthy human controls

	***L. major*** 102 (%)	Healthy human controls 199 (%)	***P*** value	OR (95%CI)
**TNF-α 308 G/A**				
Low (GG)	60 (58.8)	130 (65.3)		
High (GA + AA)	42 (41.2)	69 (34.7)		
**INF-γ 874 A/T**				
Low (AA)	28 (27.5)	76 (38.2)		
High (AT+TT)	74 (72.5)	123 (61.8)		
**TNF-α with INF-γ**				
Low/ low	18 (17.6)	51 (25.6)	0.1	0.6 (0.3 to1.1)
Low/ high	42 (41.2)	89 (44.7)	0.5	0.8 (0.5 to 1.4)
High/ low	10 (9.8)	25 (12.6)	0.4	0.7 (0.3 to 1.6)
High/ high	32 (31.4)	34 (17.1)	P** 0.005	2.2 (1.2 to 3.8)
	***L. tropica*** **59 (%)**	**Healthy human controls ** **199 (%)**		
**TNF-α 308 G/A**				
Low (GG)	35 (59.3)	130 (65.3)		
High (GA + AA)	24 (40.7)	69 (34.7)		
**INF-γ 874 A/T**				
Low (AA)	18 (30.5)	76 (38.2)		
High (AT+TT)	41 (69.5)	123 (61.8)		
**TNF-α 308 / INF-γ 874**				
Low/ low	15 (25.4)	51 (25.6)	0.7	0.8 (0.3 to 1.7)
Low/ high	20 (33.9)	89 (44.7)	0.1	0.6 (0.3 to 1.1)
High/ low	3 (5.1)	25 (12.6)	0.1	0.3 (0.1 to 1.3)
High/ high	21 (35.6)	34 (17.1)	P** 0.003	2.7 (1.4 to 5.1)
	**Total*leishmania*cases 169 (%)**	**Healthy human controls** **199 (%)**		
**TNF-α 308 G/A**				
Low (GG)	100 (59.2)	130 (65.3)		
High (GA + AA)	69 (40.8)	69 (34.7)		
**INF-γ 874 A/T**				
Low (AA)	49 (29.0)	76 (38.2)		
High (AT+TT)	120 (71.0)	123 (61.8)		
**TNF-α 308 / INF-γ 874**				
Low/ low	36 (21.3)	51 (25.6)	0.3	0.7 (0.4 to 1.2)
Low/ high	64 (37.9)	89 (44.7)	0.1	0.7 (0.4 to 1.1)
High/ low	15 (8.9)	25 (12.6)	0.2	0.6 (0.3 to 1.3)
High/ high	54 (31.9)	34 (17.1)	P** 0.001	2.2 (1.2 to 3.7)

P** highly significant

## Discussion

This study determined the association of the two important gene polymorphisms in Saudi patients with cutaneous leishmaniasis. In one of our previous studies, we reported the prevalence of *L. species* among CL patients in Qassim area of Saudi Arabia [5]. Our reported data clearly pointed out that CL patients in this region were mainly infected by *L. major* and *L. tropica* [5]. It is now well established that the parasite of leishmaniasis is endemic in all over the globe and now it becomes the major public health problem [5]. In Saudi Arabia, the prevalence of CL infection is on the rise and now it remains a major unsolved health problem of the country [22]. Several previous studies reported an association between the susceptibility and resistance of leishmaniasis with the number of cytokines gene polymorphisms [23–25], but none of the study did not show direct association of cytokines gene polymorphism with the CL infection nor with the any of the *L. species* and the controversial role of their SNPs with leishmaniasis remains continued [22–25]. The role of Th1 cytokines such as TNF-α, IFN-γ was well studied in CL patients, as their levels were found to be higher in these patients [26, 27]. In support of these, Akhzari et al. found that the expression of these cytokines in CL lesion varies with the treatment response [27]. Furthermore, Wilhelm et al. reported that treatment of CL patients with TNF-α improves the parasitic burden and decreased the CL lesion size [28]. Because of these important implications of these Th1 cytokines for CL patients and frequency of occurrence of CL infection in Saudi Arabia, this study was designed to investigate the gene polymorphisms of TNF-α-308 G/A and IFN-γ + 874 A/T in patients of CL with different *L. species* in central region of Saudi Arabia.

Analysis of TNF-α-308 G/A polymorphism on 169 Saudi patients with different *L. species* and 199 healthy controls from the same area, showed no significant association of TNF-α-308 genotype with CL patients. These results were fully supported with the other studies of TNF-α-308 polymorphism performed on different population of CL patients [28, 29]. In contract of these results, our data also showed the CL patients infected with *L. tropica*, showed significantly higher TNF-α-308 A versus G allele frequency. This may be due the different genetic backgrounds of Saudi populations. More specifically the frequency of TNF-α A allele in the Saudi healthy controls was found in a range of 18.3%, which seem to be statically significant as compared with other population such as Iranian controls (12.6%), Brazilian controls (12.2%) and Venezuelan controls (7%) [23, 29].

Besides these, this study also demonstrated IFN-γ + 874 A/T polymorphism on the same CL samples obtained from Qassim region of Saudi Arabia. Our results showed that IFN-γ + 874 genotyping (TT versus AA+AT recessive) and allelic frequency of IFN-γ + 874 (T versus A) showed significant higher in CL patients infected with *L. major* and also similar results were obtained in total CL cases as compared with their respective control humans. These are novel findings which have not been reported before. The IFN-γ + 874 TT polymorphism was reported to be associated with the transcription of IFN-γ gene in host CL, in which T allele was found to be involved in higher production of IFN-γ [17]. In addition, the resistance role of IFN-γ as well as INF-γ 874 A/T in immuno-response against leishmaniasis has also been reported in different animal models [30, 31]. All these data either directly or indirectly supported our results. Furthermore, a study by Kamali-Sarvestani et al. also supported our results by pointing out a significantly higher frequency of T allele or TT genotype of IFN-γ + 874 A/T polymorphism in patients infected with *L. major* in Iranian population [29]. In contrast of these results, Matos et al. reported no association between IFN-γ + 874 A/T polymorphism with the susceptibility leishmaniasis [32]. Despite of these, IFN-γ + 874 A/T SNP seems to be involved in the pathogenesis of leishmaniasis by influencing the amount of cytokine released in CL patients [32]. In support of these, Al-Bushier reported an association of IFN-γ + 874 A/T polymorphism with the susceptibility of visceral leishmaniasis [33]. To further determine the potential of TNF-α 308 G/A and INF-γ + 874 A/T SNPs in the susceptibility of CL infection, the analysis was performed on the synergistically combined TNF-α 308 G/A and INF-γ + 874 A/T alleles. The calculated data showed strong association between the combined TNF-α-308 G/A and IFN-γ + 874 A/T gene polymorphisms with the susceptibility of *L. major*, *L. tropica* and also with total studied CL patients. In short, the data demonstrated no association of TNF-α-308 gene polymorphism with the susceptibility of CL in Saudi patients. The only association was found in between A versus G allelic frequency in *L. tropica*. Whereas, IFN-γ + 874 A/T polymorphism was found to be associated with the susceptibility of *L. major* and also with the total studied CL cases. Moreover, the data from the synergistically combined high TNF-α 308/INF-γ 874 alleles strongly suggested their potential role in the susceptibility of *leishmania* infection.

## Conclusions

This is the first report that shows the two most important gene polymorphisms of TNF-α-308 G/A and IFN-γ + 874 A/T in Saudi patients infected with different *L. species*. The study demonstrated no association between the host TNF-α-308 G/A gene polymorphism and CL infection in Saudi patients. The only significant correlation was found in A versus G allelic frequency in CL patients infected by *L. tropica*. Interestingly, IFN-γ-874 A/T polymorphism was found to be significantly correlated with the susceptibility of *L. major* and also in total *leishmania* subjects, suggesting its role in the production of IFN-γ, and thus enhance immune protection. Furthermore, a notable association between the susceptibility of *L. major*, *L. tropica* or total studied CL patients with the synergistically combined high TNF-α 308/INF-γ 874 alleles, strongly suggested their crucial role in the onset of CL infection among the studied Saudi population.

## Supplementary information

**Additional file 1.**

## Abbreviations

CL: Cutaneous leishmaniasis
*L. major*: *Leishmania major*
*L. tropica*: *Leishmania tropica*
*L. infantum/donovani*: *Leishmania infantum/donovani*
TNF-α: Tumor necrosis factor alpha
IFN-γ: Interferon gamma
Th1 cells: Type 1 T helper cells
KACST: King Abdulaziz City for science and technology
NSTIP: National plan for science, technology and innovation
